# A moderated mediation analysis of the association between smoking and suicide attempts among adolescents in 28 countries

**DOI:** 10.1038/s41598-023-32610-8

**Published:** 2023-04-08

**Authors:** Prince Peprah, Bernard Yeboah-Asiamah Asare, Reforce Okwei, Williams Agyemang-Duah, Joseph Osafo, Irene A. Kretchy, Razak M. Gyasi

**Affiliations:** 1 Social Policy Research Centre, University of New South Wales, Sydney, Australia; 2 Centre for Primary Health Care and Equity, University New South Wales, Sydney, Australia; 3 Curtin School of Population Health, Curtin University, Kent Street, Bentley, WA 6102 Australia; 4 Institute of Applied Health Sciences, University of Aberdeen, Aberdeen, AB25 2ZD UK; 5 Department of Geography, Miami University, Oxford, USA; 6 Department of Geography and Planning, Queen’s University, Kingston, ON K7L 3N6 Canada; 7 Department of Psychology, University of Ghana, Legon, Ghana; 8 Centre for Suicide and Violence Research - CSVR, Accra, Ghana; 9 Department of Pharmacy Practice and Clinical Pharmacy, School of Pharmacy, College of Health Sciences, University of Ghana, Legon, Ghana; 10 African Population and Health Research Center, Manga Close, Off-Kirawa Road, P.O. Box 10787, Nairobi, 00100 Kenya

**Keywords:** Psychology, Medical research, Risk factors

## Abstract

Globally, evidence has shown that many adolescents are victims of substance use, mainly cigarette smoking, and it has been associated with suicidal ideation. However, the mechanisms underlying this association are poorly understood. This study examines whether truancy mediates and gender moderates the association of cigarette smoking with suicide attempts among adolescents in 28 countries. Data from the Global School-Based Student Health Survey were used. Hierarchical multiple logistic regression analyses were used to estimate the effect-modification of gender on cigarette smoking and suicide attempt. The mediating effect of truancy on the association between cigarette smoking and suicidal attempt was assessed using the generalized decomposition method. Cigarette smoking was associated with suicide attempts after adjusting for several confounding variables (aOR = 1.21; 95% CI = 1.09–1.33). The bootstrap results from the generalized decomposition analysis indicated that truancy partially mediated the association of cigarette smoking with a suicide attempt, contributing 21% of the total effect among in-school adolescents. Hierarchical regression analyses suggested that gender moderated the effect of cigarette smoking on suicidal attempts: female adolescents who smoked had 36% higher odds of suicidal attempts compared to male adolescents. The findings suggest possible pathways for designing and implementing interventions to address adolescents' cigarette smoking and truancy to prevent suicidal attempts.

## Introduction

Cigarette smoking has become an epidemic with attendant adverse outcomes on health and healthcare systems globally^1,2^. Previous research has indicated that many adolescents, including school-going adolescents, use substances like cigarettes^3,4^. Studies have shown that a median of 22.7% of adolescents in school had ever smoked cigarettes, and 15.4% had attempted marijuana before attaining 13 years^5^. Also, the Global Youth Tobacco Surveys among 143 countries have revealed that 11.3% and 6.1% of boys and girls, respectively, reported smoking cigarettes at least one day during the past 30 days^6^. The usage and abuse of substances, like cigarettes, have been associated with suicidal behaviour^7–10^. Suicide behaviours comprise mindsets, efforts, and loss of life by suicide^11^. Suicide is a significant issue in public health discussions, mainly due to the evidence that it is the second leading cause of death among youthful populations between the ages of 10 and 24 years globally^12^. Also, among persons aged 15 to 19, suicide is the third contributor to the cause of death^13^. Studies suggest that cigarette smokers are more likely to attempt suicide than non-smokers ^14–16^. In a longitudinal study, McGee et al.^9^ found that early smoking predicted later suicidal ideation among adolescents. Similarly, a recent study among high school students by Dasagi et al.^17^ found that cigarette smokers were almost three times more likely to report suicidal ideations and two times more likely to plan a suicide attempt than non-smokers.

Although research has documented a significant association between cigarette smoking and suicide^18^, age and gender, have been identified as proximate factors that exacerbate the risk, exposure, and increasing propensity of suicidal behaviors^19^. Nevertheless, the mechanism of how smoking cigarettes may lead to suicidal attempts and whether this varies with gender differences is still unknown. Meanwhile, factors such as truancy could potentially serve as a link between the smoking-suicide association^20,21^. In adolescents, truancy has been identified as a significant risk factor for short-term and long-term negative psychological consequences like suicidal behaviours^22,23^. Evidence has linked truancy to mental health problems such as depression, anxiety, and suicide behaviours^24^. A recent study found that truancy was a predictor of suicide behaviours^25^. At the same time, as one of the most relevant predictors, cigarette smoking significantly contributes to adolescents’ truancy experience. Evidence from several studies has demonstrated the link between smoking and truancy^26,27^. For example, a recent study using data from the Global School-Based Student Health Survey in Liberia found that cigarette smoking increased the risk of truancy among adolescents^26^. Another study also indicated that adolescent truancy might be associated with cigarette smoking^28^. From previous studies, it is plausible that truancy may mediate the association between cigarette smoking and suicide attempts among in-school adolescents. However, previous research has least explored this critical pathway. Thus, this study seeks to address this research gap.

Similarly, considerable empirical and theoretical support exists for the view that there are some significant disparities between the suicidality of males and females^29–32^. Studies note that gender differences may exist in smoking-related suicide attempts^30–32^. This observation is due to the view that smoking is more rampant among males than females, especially in low- and middle-income countries, where the cultural setting frowns more on female smokers than male smokers. This assertion could be a proximal cause of the potential gender disparities in smoking induced-suicide attempts. It has been suggested that males and females can manage and deal with their emotions differently^33^, and females are less likely to manage suicide triggers like stress, anxiety, and depression effectively^34^. It has also been explained that though males may feel depressed and hopeless, they are less likely to admit and seriously consider suicide than females due to the perception that suicidal ideation is a sign of weakness and inadequacy in managing one’s affairs^35^. However, while gender could affect suicide attempts and gender differences may exist in the relationship between cigarette smoking and suicide attempts, the modifying role of gender in this relationship has not been explored. For example, McGee et al.^9^, in their study assessing the association between cigarette smoking and suicidal ideation, did not examine the modifying role of gender. With prior studies prompting the inclusion of gender into studies on suicidality, this study, therefore, examines the moderation effect of gender in the association between cigarette smoking and suicidal attempts among in-school adolescents.

### The present study

To the best of the authors’ knowledge, the mediation effect of truancy and moderating effect of gender on the association between cigarette smoking and suicidal attempts is limited. The study tests two hypotheses to address this knowledge gap. First, truancy mediates the effect of in-school adolescents’ cigarette smoking on suicidal attempts. Second, gender would moderate the association between cigarette smoking and suicidal attempts among school-going adolescents. The study provides evidence on possible pathways for initiating, designing, implementing, and evaluating programs, interventions, and services to address adolescents’ cigarette smoking and truancy to prevent suicidal attempts. It also suggests the importance of gender consideration in implementing cigarette smoking and truancy prevention.

## Methods

### Data and participants

This study used data from the Global School-based Student Health Survey (GSHS) among 28 countries and territories. The GSHS is a large and representative survey of students' health behaviours and risk factors. The survey was developed by the World Health Organization (WHO) and the US Centers for Disease Control and Prevention (CDC), and other United Nations (U.N.) allies and country-specific institutions^36,37^. The survey draws content from the CDC Youth Risk Behaviors Survey (YRBS), for which test–retest reliability has been established^38^. Information, including the aims, methodology, and sampling procedure of the GSHS, is available at http://www.cdc.gov/gshs/.

In summary, the participants for the GSHS are primarily school-going adolescents aged 13–17 years. The sampling procedure included a standardized two-stage probability sampling approach for the participant selection procedure within each participating country. In the first stage, the schools were selected through probability proportional to size sampling design. The second stage involved randomly selecting classrooms, primarily including students aged 13–17 in each selected school. Irrespective of age, all students in the selected classrooms were eligible to participate in the survey.

**Figure 1:**
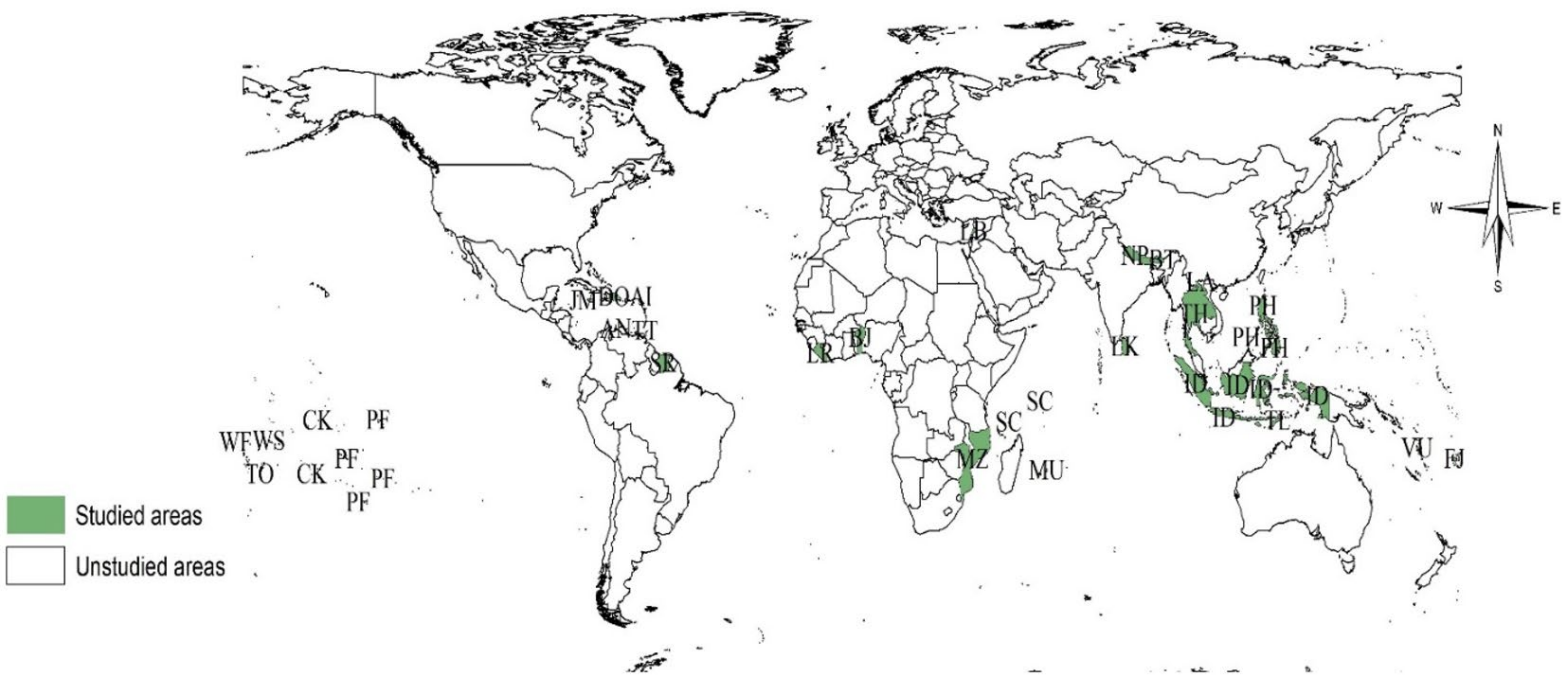
World map showing the study locations (Produced within ArcGIS version 10.8.1 using a base map obtained from https://thematicmapping.org/downloads/world_borders.php). Note: Anguilla (A.I.), Benin (B.N.), Bhutan (B.T.), Cook Islands (C.K.), Curacao (CC), Dominican Republic (D.O.), Fiji (F.J.), French Polynesia (P.F.), Indonesia (I.D.), Jamaica (J.M.), Lao People’s Democratic Republic (L.A.), Lebanon (L.B.), Liberia (L.R.), Mauritius (M.U.), Mozambique (M.Z.), Nepal (N.P.), Philippines (P.H.), Samoa (W.S.), Seychelles (S.C.), Sri Lanka (S.L.), Saint Lucia (L.C.), Suriname (S.R.), Thailand (T.H.), Timor-Leste (T.L.), Trinidad and Tobago (T.T.), Tonga (TO), and Vanuatu (V.U.), Wallis and Futuna Islands (W.F.).

This study used the most recent data available in GSHS-participating countries and territories. Countries and territories with their most recent data released before 2015 were excluded to ensure that the analysis reflects and represents recent or current trends. As a result, 23 countries were excluded because data were taken before 2015, and 12 other countries have also excluded the variables of interest for the present study were missing from their datasets. By applying the inclusion and exclusion criteria, data from 28 countries and territories were considered recent and eligible to be included in the analysis. Thus, our analysis included data from 28 countries and territories released between 2015 and 2018. Data were weighted for non-response and probability selection to generalize results to the eligible population^39,40^. The complete data on all variables included in the analysis for this study was available for 16,213 adolescents. The characteristics of each country and territory, including the country/territory name, survey year, and sample size, are provided in Table 1. The location of the included studies is also shown in Fig. 1.

**Table 1:** Distribution of study sample across countries and territories.

Country	Year of survey	Weighted N	Weighted (%)
Anguilla	2016	80	0.5
Benin	2016	179	1.1
Bhutan	2016	2213	13.7
Cookes Island	2015	274	1.7
Curacao	2015	632	3.9
Dominican Republic	2016	149	0.9
Figi	2016	613	3.8
French Polynesia	2015	1113	6.9
Indonesia	2015	1760	10.9
Jamaica	2017	407	2.5
Lao Republic	2015	259	1.6
Lebanon	2017	902	5.6
Liberia	2017	155	1.0
Maurituis	2017	631	3.9
Mozambique	2015	49	0.3
Nepal	2015	374	2.3
Philippines	2015	1513	9.3
Samoa	2017	194	1.2
Seychelles	2015	628	3.9
Sri Lanka	2016	136	0.8
St Lucia	2018	262	1.6
Suriname	2016	466	2.9
Thailand	2015	711	4.4
Timor Leste	2015	495	3.1
Trinidad and Tobago	2017	627	3.9
Tonga	2017	621	3.8
Vanuatu	2016	395	2.4
Wallis and Futau	2015	375	2.3
Total		16,213	100.0

### Procedure

Data collection was conducted during an average class period with the help of trained enumerators from local social work organizations and other research institutions. The GSHS questionnaire was close-ended and structured, developed in English but translated into the local language in each country. The questionnaire included multiple-choice questions. After informed consent was obtained from the students, parents, and school officials, the questionnaires were administered. The trained enumerators explained the aim of the study and the instructions to the students before the questionnaires were distributed. In addition, the survey ensured that students’ privacy was protected through anonymous and voluntary participation. The study complied with the Declaration of Helsinki. Each participating country’s institutional review board or ethics committee reviewed and approved the GSHS survey. Also, details of the systematic procedures used for the data collection among the students can be found at http://www.cdc.gov/gshs/.

### Measures

#### Cigarette smoking (Exposure variable)

We assessed cigarette smoking using the question, “During the past 30 days, on how many days did you smoke cigarettes? (0 days, 1 or 2 days, 3 to 5 days, 6 to 9 days, 10 to 19 days, 20 to 29 days and, All 30 days)”. This study considered cigarette smoking at least one day in the past 30 days. Those who answered 0 days were considered non-smokers, and those who responded one or more days were considered cigarette smokers. The dichotomization was informed by the fact that responses were highly skewed^41^.

#### Suicidal attempt (Outcome variable)

The suicidal attempt was measured using the item: “During the past 12 months, how many times did you attempt suicide? (0, 1, 2, 3, 4, 5 and 6 or more)”. Suicidal attempt in this study was defined as at least one attempt in the past 12 months as used in previous studies^42–44^. Respondents who answered 0 attempts were considered as having experienced no suicide attempt and coded as “0”, and those with one or more attempts were considered as having experienced a suicide attempt and coded as “1”.

#### Truancy (Mediator)

We assessed truancy with the item: “During the past 30 days, how many days did you miss classes or school without permission? (“0 days, 1 or 2 days, 3 to 5 days, 6 to 9 days and ten or more)”. It was dichotomized into “1”(if the respondent missed school at least one day without permission) and “0” (if the respondent did not miss school without permission). The outcome variable was recoded on a dichotomous scale, consistent with previous studies on truancy using GSHS data^23,25,45–47^.

#### Gender

Gender was included as the moderator in this study and was treated as a binary variable with “0” if the participant identified as female and “1” if the participant identified as male.

#### Control variables

Participants self-reported all demographics and health-related variables such as age, alcohol intake, drug use, physical activity, lack of sleep, parental involvement, physical violence, bullying, and loneliness were included in the regression models as confounders. The health-related variables included were based on evidence of their association with smoking and suicide attempts in previous studies among adolescents^25,48^ and their availability in the dataset. The classification of these measures is provided in Table 1.

### Statistical analysis

Descriptive statistics were calculated to describe the socio-demographic characteristics. The Chi-square test examined the differences in suicidal attempts between socio-demographic and health-related variables. Hierarchical multivariable logistic regressions were performed to examine the association between cigarette smoking and suicidal attempts. The moderation effect of gender on the association between cigarette smoking and the suicidal attempts was examined in three steps; in step 1 (model 1), we specified a model to examine the main effects of gender on suicidal attempts; in step 2 (model 2), we tested the independent effects of gender and cigarette smoking on suicidal attempts; and in step 3 (model 3), an interaction between cigarette smoking and gender (cigarette smoking × gender) was included to estimate an effect modification of the association by gender. The mediating effect of truancy on the association between cigarette smoking and suicidal attempts was assessed using the generalized decomposition method in the *ldecomp* package in Stata^41^. *ldecomp* method decomposes the total effect in a logit model into direct and indirect effects. It allows variables through which the indirect effect occurs (mediators) to have any distribution and uses bootstrapping to estimate odds ratios, standard errors, and confidence intervals. It specifies the direct, indirect, and total effects and the average contributing effect of the mediator (indirect effect) to the total effect^41^. All statistical analyses were performed with STATA version 13 software (StataCorp L.P., College Station, TX, USA). All statistical significance was set at ≤ 0.05.

## Results

### Background information of the study participants

The study included a sample of 16,213 school-going adolescents. Most respondents were aged between 15 and 17 years (56.8%), and 61.4% were males. More than half of the respondents participated in physical activity (54.6%), while 45.9% used alcohol and 15.9% used other drugs. Table 2 presents the background information of the study participants.

**Table 2:** Description of sample (n = 16,213).

Characteristics	Total, n (%)	Suicide attempt, n (%)	*p* value
Age (years)			< 0.001***
< 15	4832 (29.8)	872 (18.0)	
15–17	9216 (56.8)	1459 (15.8)	
18 years and more	2165 (13.4)	313 (14.5)	
Gender			< 0.001***
Male	9955 (61.4)	1152 (11.6)	
Female	6258 (38.6)	1492 (23.8)	
Smoking			
No	7937 (49.0)	1078 (13.6)	< 0.001***
Yes	8276 (51.0)	1566 (18.9)	
Alcohol intake			< 0.001***
No	8774 (54.1)	1145 (13.0)	
Yes	7439 (45.9)	1499 (20.2)	
Drug use			< 0.001***
No	13,635 (84.1)	2000 (14.7)	
Yes	2578 (15.9)	644 (25.0)	
Physical activity			0.057
No	7361 (45.4)	1245 (16.9)	
Yes	8852 (54.6)	1399 (15.8)	
Sleep disturbances			< 0.001***
No	8437 (52.0)	921 (10.9)	
Yes	7776 (48.0)	1723 (22.2)	
Parental involvement			< 0.001***
No	2553 (15.8)	533 (20.9)	
Yes	13,660 (84.2)	2111 (15.4)	
Experience physical violence			< 0.001***
No	7375 (45.5)	845 (11.5)	
Yes	8838 (54.5)	1799 (20.4)	
Bullying			< 0.001***
No	11,749 (72.5)	1427 (12.2)	
Yes	4464 (27.5)	1217 (27.3)	
Loneliness			< 0.001***
No	7793 (48.1)	853 (11.0)	
Yes	8420 (51.9)	1791 (21.3)	
Truancy			< 0.001***
No	10,183 (62.8)	1387 (13.6)	
Yes	6030 (37.2)	1257 (20.9)	

****p < ; ***p < 0.001; **p < 0.01; *p < 0.05.

### Prevalence of suicidal attempts, cigarette smoking, and truancy

**Figure 2:**
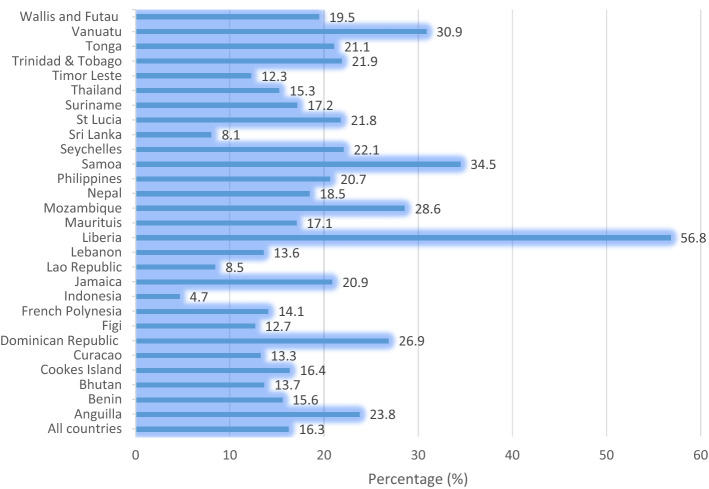
Prevalence of suicidal attempts across the 28 countries and territories.

**Figure 3:**
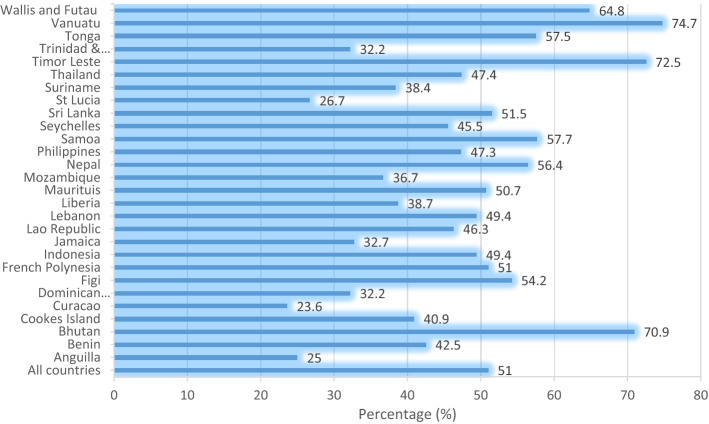
Prevalence of cigarette smoking across the 28 countries and territories.

**Figure 4:**
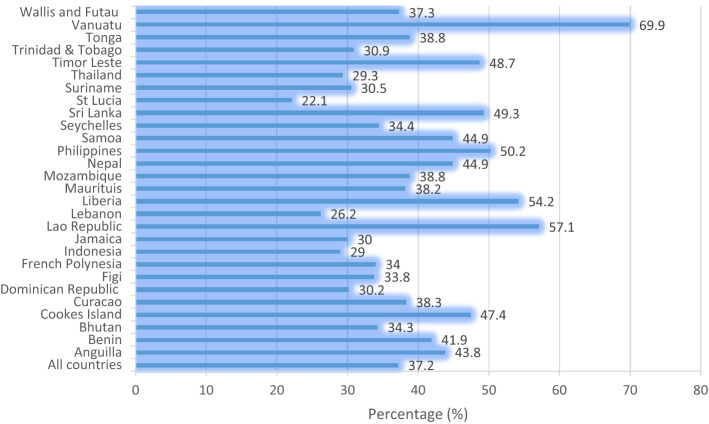
Prevalence of truancy across the 28 countries and territories.

The prevalence of suicide attempts, cigarette smoking, truancy, and suicidal attempts across the 28 countries and territories are shown in Figs. 2, 3, and 4. The overall prevalence of suicidal attempts across the 28 countries and territories was 16.3%. Liberia had the highest prevalence (56.8%), whereas Indonesia reported the lowest (4.7%) (Fig. 2). The results indicated that overall, 51% of the adolescents smoked cigarette; Vanuatu had the highest prevalence (74.7%), whereas Cuaracao reported the lowest (23.6%) (Fig. 3). The overall prevalence of truancy was 37.2%, and at the country level, Vanuatu recorded the highest prevalence (38.3%), whereas the lowest (22.1%) was recorded in St Lucia (Fig. 4).

### The mediating effect of truancy on the association between smoking status and suicidal attempt

The mediation analysis revealed a positive direct relationship between cigarette smoking with suicidal attempts (OR = 1.21; 95% CI = 1.09–1.33, *p* < 0.000) (Table 3). The bootstrap results from the generalized decomposition analysis showed that the mediating effect of truancy was 1.05, with a 95% confidence interval of [1.04, 1.07]. Specifically, the odds of attempting suicide among adolescents who smoked cigarette were 1.27 times as large as the odds for adolescents who were not smoking (i.e., the total effect). Adolescents who did not smoke cigarette were at 1.05 times higher odds of attempting suicide if they reported truancy than adolescents who smoked cigarette (i.e. indirect effect). In contrast, adolescents who smoked cigarette had 1.21 times higher odds of attempting suicide than adolescents who did not smoke cigarette when truancy is kept constant at the rate reported for those who smoke (i.e. direct effect). On average, the indirect effect (of truancy) accounted for 21% of the total effect of cigarette smoking on suicidal attempts (Table 3). This finding indicates that truancy partially mediated the association between smoking status and suicidal attempts among school-going adolescents.

**Table 3:** Bootstrap results of the decomposition analysis estimating the mediating effect of truancy on the association between cigarette smoking and suicidal attempts.

	Observed OR	Bootstrap Std. Err	z	*P* value	Normal-based 95% CI
2/1					
Total	1.268	0.062	4.87	0.000****	1.15, 1.39
Indirect1	1.051	0.008	6.30	0.000****	1.04, 1.07
Direct1	1.206	0.059	3.79	0.000****	1.09, 1.33
Indirect2	1.051	0.008	6.31	0.000****	1.04, 1.07
Direct2	1.206	0.060	3.79	0.000****	1.09, 1.33
2/1r					
Method1	0.211	0.062	3.41	0.001***	0.09, 0.33
Method2	0.211	0.062	3.40	0.001***	0.09, 0.33
Average	0.211	0.062	3.40	0.000****	0.09, 0.33

2/1: the sizes of direct, indirect, and total effects of current smoking on suicide attempt.

direct1: direct effect of current smoking according to decomposition method 1.

direct2: direct effect of current smoking according to decomposition method 2.

indirect1: represent the indirect effect of current smoking according to decomposition method 1.

indirect2: represent the indirect effect of current smoking according to decomposition method 2.

2/1r: the sizes of the indirect effects relative to the total effects.

Adjusted for age, gender, alcohol intake, drug use, physical activity, lack of sleep, parental involvement, physical violence, bullying, loneliness.

****p < ; ***p < 0.001; **p < 0.01; *p < 0.05.

### The moderating effect of gender on the association of cigarette smoking with suicidal attempts

We performed a moderation analysis to examine the interaction effect of gender on the association between smoking and suicidal attempts (Table 4). The independent effects of smoking (OR = 1.21, 95%CI = 1.09–1.33) and gender (being a female) (OR = 2.50, 95%CI = 2.28–2.75), after adjusting for age, alcohol intake, drug use, physical activity, lack of sleep, parental involvement, physical violence, bullying, and loneliness, were associated with higher odds of suicidal attempts (Model 2). In adding the interactive term (smoking x gender) in model 3, results showed that the independent association between smoking and suicidal attempts was nullified (OR = 1.02, 95%CI = 0.89–1.17, *p* > 0.05), and gender moderated the association. Among the adolescents who smoked, those who were females had 36% higher odds of suicidal attempts compared with their male counterpart (OR = 1.36, 95%CI = 1.14–1.63).

**Table 4:** Hierarchical regression models assessing the interaction between gender and cigarette smoking on suicidal attempts.

Characteristics	Model 1 OR (95%CI)	Model 2 OR (95%CI)	Model 3 OR (95%CI)
Gender			
Male	1	1	1
Female	2.46 (2.24–2.70)***	2.50 (2.28–2.75)***	2.10 (1.83–2.41)***
Smoking status			
No		1	1
Yes		1.21 (1.09–1.33)***	1.02 (0.89–1.17)
Smoking x gender (male = 0, female = 1)			1.36 (1.14–1.63)**
*R*^2^	0.1125	0.1135	0.1143
*Log-likelihood*	− 6399.496	− 6392.2433	− 6386.6889
*Prob* > *chi2*	0.0000	0.0000	0.0000

Model 1 tested the independent effect of gender on suicidal attempts; Model 2 tested the independent effects of gender and smoking on suicidal attempts; Model 3 tested the interaction between gender and smoking on suicidal attempts.

All model adjusted for age, alcohol intake, drug use, physical activity, lack of sleep, parental involvement, physical violence, bullying, loneliness, truancy.

***p < 0.001; **p < 0.01; *p < 0.05.

## Discussion

In this multi-setting study, we investigated the associations between cigarette smoking and suicide attempts and tested the mediation and moderation effects of truancy and gender on the association. The prevalence of cigarette smoking and suicidal attempts across the 28 countries was 51% and 16.3%, respectively. Cigarette smoking was found to influence suicidal attempts; adolescents who smoked were more likely to attempt suicide. This finding is consistent with previously published studies that found associations between cigarette smoking and suicidal attempts among adolescents^22–49^. Waters et al.^50^ have found a dose–response association between smoking and suicidal ideation, such that the higher the smoking rate, the greater the suicidal ideation, even after controlling for depression, alcohol use and drug use. Lange and colleagues^51^ have reported a positive association between smoking and suicidal attempts among adolescents aged 12-15 years regardless of country and gender. For girls, a dose–response relationship between cigarette smoking and secondhand smoke exposure with suicidal attempts existed. Cigarette smokers are more likely to report higher levels of stressful events, anxiety, depressive symptoms, and more chronic diseases than those who are non-smokers^51^. Thus, cigarette smoking is a significant predictor of suicidal attempts among adolescents. This evidence suggests that addressing cigarette smoking could help reduce the psychological issues, such as stress, anxiety, depression, and tension associated with suicidal ideation^51^.

Our results showed that the cigarette smoking-suicidal attempts relationship was mediated by the truancy path, supporting our second hypothesis that cigarette-smoking adolescents are more likely to report suicidal attempts via truancy. Truancy explained 21% of the effect of smoking on suicidal attempts among in-school adolescents. This study is the first to disentangle the complex interrelationships between cigarette smoking, truancy and suicidal attempts using large-scale and multi-country data among adolescents. Our findings contribute to the extant literature on improving the understanding of the pathways underlying the association between cigarette smoking and suicide behaviours among adolescents. This finding is consistent with earlier studies indicating that cigarette smoking was a risk factor for truancy^26,27^ and a predictor of suicidal attempts^25^. Adolescence is a fragile stage of growth, and evidence shows that adolescents who smoke cigarettes due, in part, to limited parental supervision and peer influence become exposed to various deviant behaviours, including truancy^52–55^. Adolescents may use truant behaviour as a coping strategy to escape from perceived undesirable environments that cause stress and anxiety, which in turn, are more prone to risky behaviours, including cigarette smoking. At the same time, truancy may lead to various physical and psychological disorders such as bullying victimization, injuries, loneliness, stress, depression, and anxiety leading to suicidal attempts^22,23^. For example, Burton et al.^24^ found that truancy in the form of absenteeism was associated with depression and anxiety symptoms among adolescents in the US, which are known risk factors for suicidal attempts. In addition, a cross-sectional study among in-school adolescents from five Southeast Asian countries has established truancy to be associated with suicide attempts in the past 12 months^52^. Moreover, numerous global studies suggest that truant behaviour is strongly associated with suicidal attempts among adolescents^56,57^. Although these associations may be bidirectional, adolescents who smoke can be truant and experience various psychological issues, promoting their risk of suicidal attempts. Dynamic strategic approaches involving all stakeholders are essential to tackle truancy in school to address suicide attempts^58^ Further longitudinal studies are also required to infer causal relationships between cigarette smoking, truancy and suicidal attempts.

This study found a moderating effect of gender on the association between smoking and suicidal attempts, consistent with our hypothesis and previous studies^29–32,59,60^. Specifically, our study found that female adolescents who were current smokers had 36% higher odds of suicidal attempts compared with their male counterpart. Our finding indicates that female adolescents are more susceptible to psychological maladjustment after smoking cigarettes and partly confirms the gender paradox hypothesis proposed by other studies^61^. Considerable empirical and theoretical supports exist for the view that significant disparities exist between the suicidality of males and females^29–32^. Studies note that gender differences may exist in smoking-related suicide attempts^30–32^. This observation is due to the view that smoking is more rampant among males than females, especially in low- and middle-income countries, where the cultural setting frowns more on female smokers than male smokers. This assertion could be a proximal cause of the potential gender disparities in smoking induced-suicidal attempts. It has been suggested that males and females can manage and deal with their emotions differently^33^, and females are less likely to manage suicide triggers like stress, anxiety, and depression effectively^34^. It has also been explained that though males may feel depressed and hopeless, they are less likely to admit and seriously consider suicide than females due to the perception that suicidal ideation is a sign of weakness and inadequacy in managing one’s affairs^35^. Our finding, therefore, implies that gender cannot be overlooked in the design and implementation of policies and interventions to address cigarette smoking and suicide attempts. In this context, school-based suicidal ideation prevention programs and interventions should be developed and implemented through a gender lens. Also, this study did not perform slope analysis to visually investigate the interaction between cigarette smoking and gender differences in predicting the likelihood of suicide attempts. Therefore, it suggests further studies may consider conducting this critical analysis.

There are strengths and limitations to the present study. Drawing evidence from 28 countries/territories, this study contributes to the limited knowledge base by examining the mediation effects of truancy and the moderation effect of gender on the association between cigarette smoking and suicidal attempts among school-going adolescents. Our sample used nationally representative datasets with a large sample size across several countries and territories, increasing the study findings' generalizability. The survey used rigorous probability sampling strategies to select and recruit nationally representative samples, indicating that our results can be generalized to other persons and countries with similar characteristics to the adolescents and countries included in this study. Also, the GSHS followed the best standard practices regarding technique and employed professional and well-trained interviewers.

Despite these strengths, our results should be interpreted and viewed in the context of the following limitations. Although we adjusted for most known potential confounders, it is still possible that residual confounding may exist that impact or explain the results. Also, relying on self-reports suggests that participants’ recall and social desirability biases cannot be ruled out. In addition, in line with previous studies, the timeframe for assessment of cigarette smoking (past 30 days) and suicide attempts (past 12 months) differed. However, in this cross-sectional study which does not infer a causal relationship between smoking and suicide attempts, it is possible to evaluate the effect of in-school adolescents’ cigarette smoking on their suicidal attempts. It is also possible that among adolescents who smoked cigarettes in the past 30 days, most also smoked cigarettes in the previous year^61^. In addition, notable studies using the same GSHS data used the past 30 days and 12 months’ timeframe to assess exposure and outcome variables^43,62–64^.

Moreover, our analysis may be limited by possible endogeneity (concern over reverse causality/simultaneity) since suicidal attempts may also affect cigarette smoking and truancy. However, a robust theoretical argument/support exists for a possible interrelationship between cigarette smoking, truancy, and suicidal attempts. Lastly, given that this study was cross-sectional, causal relationships and implications from the association between smoking and suicidal attempts cannot be inferred. Thus, future studies should examine the associations of interest using longitudinal cohort designs that enable the investigation of plausible causal inferences. Also, cigarette smoking and gender could be highly correlated, as men are more likely to smoke than women, but was not examined in this study. Again, country-level variables, such as per capita GDP growth, media freedom, and armed conflict, could impact suicide attempts. These relationships and cross-level effects could be explored in subsequent studies. Furthermore, gender could moderate the effect of cigarette smoking on truancy, which could further exert distinct effects on suicidal attempts.

## Conclusions

This multi-country study has provided evidence showing the associations between smoking and suicidal attempts and the mediating role of truancy as well as moderating role of gender, in this relationship. These findings show that addressing cigarette smoking and truancy and enhancing preventive measures and support for adolescents, predominantly female cigarette smokers of school-going age, could reduce their suicide risk behaviours. This evidence necessitates targeted human management options, preventative programs, policies, and techniques for improved public mental health and well-being among school-going adolescents.

## Data Availability

The data and necessary details to replicate the results are available at http://www.cdc.gov/gshs/.
